# Testicular tumors in the “elderly” population

**DOI:** 10.3389/fonc.2022.972151

**Published:** 2022-09-16

**Authors:** Simona Secondino, Giovanni Rosti, Antonino C. Tralongo, Franco Nolè, Domiziana Alaimo, Ornella Carminati, Richard Lawrence John Naspro, Paolo Pedrazzoli

**Affiliations:** ^1^ Department of Oncology and Hematology, Medical Oncology Unit, Istituto di Ricerca e Cura a Carattere Scientifico (IRCCS) Policlinico San Matteo, Pavia, Italy; ^2^ Department of Internal Medicine and Medical Therapy, University of Pavia, Pavia, Italy; ^3^ Medical Oncology Unit Umberto I Hospital, Rete Assistenza Oncologica (RAO) Department of Oncology, Azienda Sanitaria Provinciale (ASP), Siracusa, Italy; ^4^ Medical Oncology Division of Urogenital and Head & Neck Tumours, European Institute of Oncology Istituto di Ricerca e Cura a Carattere Scientifico, Milan, Italy; ^5^ Medical Oncology, Ospedale per gli Infermi, AUSL Romagna, Rimini, Italy; ^6^ Urology, Fondazione IRCCS Policlinico San Matteo, Pavia, Italy

**Keywords:** germ cell tumor, elderly population, seminoma, non-seminoma, old cancer patient

## Abstract

Germ cell tumors arise in childhood but peak at around 30 years of age. They are the most common cancers in males under the age of 35. Over 95% arise in the testes while a minority originate in extragonadal sites such as the anterior mediastinum, or mainly in childhood the pineal gland or the sacrococcygeal area. These tumors show an extraordinary sensitivity to chemotherapy (and for seminoma, also to radiation) and cure rates are relatively high even in second or subsequent relapses. Very few data are present in the literature regarding patients diagnosed after 50 years and no specific trials have been conducted in this setting. Nearly all patients reported in the literature had testicular cancers, with occasional reports of extragonadal tumors. Despite the fact that > 50 years may be considered an “elderly” population, these patients are treated with the same cisplatin containing combinations as their younger counterparts with consequent higher toxicity. In this review we will present epidemiological and clinical data from this rare population of patients with testicular cancer.

## Introduction

Testicular cancers account for only 1% of all tumors in males but are the most common cancer in men between 15 and 39 years of age (1).

The disease is extremely rare prepuberty (2) and its frequency declines after the age of 50 (1).

Very few data exist regarding the clinical characteristics and outcome in patients with testicular cancer after the age of 50 due to the rarity of this disease in elderly individuals.

We will try to summarize what we know regarding epidemiological, histological and clinical aspects of these tumors arising in the this population.

Usually, the term elderly in Medical Oncology refers to patients 70 years and above, but due to epidemiology of testicular tumors, we consider 50 as “elderly”.

## Epidemiology

Testicular tumors are divided into germ cell and non-germ cell cancers. The vast majority (over 95%) are of germ cell origin, and the others include sex cord stromal tumors (i.e., Leydig cell tumors and Sertoli cell tumors), together with some other extremely rare diseases (3).

Other primary neoplastic diseases originating in the testes are lymphoma, usually B cell type (3).

Germ cell tumors include seminoma and non-seminoma and the very rare spermatocytic tumors, called spermatocytic seminoma until a few years ago (3).

Seminoma is slightly more frequent than non-seminoma in patients below 50 years and it tends to develop at a later mean age (around 35 years) compared to non-seminoma. Cases of mixed histology (seminoma and non-seminoma) have to be considered as non-seminoma for therapy and prognosis despite the different percentages of the various components: teratoma, yolk-sac tumors, embryonal carcinoma, choriocarcinoma, and seminoma. Nearly 70-80% of germ cell tumors express at least one of the following tumor markers; beta-hCG, alfa-fetoprotein, LDH (4).

The incidence of germ cell tumors is increasing worldwide, albeit with marked regional differences. Very rare in Africa and among Afro-American populations and in Asia, they have a higher incidence in Nordic European counties especially Denmark, Norway and Sweden.

Nevertheless, the increase in incidence has been observed all over the world including those areas mentioned above with a lower frequency of the disease (5). It is a disease involving adult young adolescents and males in their third and fourth decades. The incidence of germ cell tumors declines markedly towards the age of 50, and tumors in patients above the age of 60 are extremely rare (6).

The mechanisms underlying this complicated epidemiological picture are not fully understood.

Information on this topic derives from the Surveillance, Epidemiology and End Results Program (SEER) Registry from National Cancer Institute of the USA (7), which reported the low incidence in patients older than 50 years. The frequency decreases even more in the group between 60-85 years old. Specifically, the incidence rate by ages at diagnosis for all races (Hispanic included), between 2014 and 2018, was 4.6 (95% CI 4.3-4.9) per 100,000 patients among 50-54 year olds; 3.4 (95%CI 3.1-3.6) per 100,000 among 55-59 year olds; 2.3 (95%CI 2.1-2.6) per 100,000 patients among 60-64 yearolds; 1.5 (95%CI 1.3-1.8) per 100,000 patients among 65-69 year olds; 1.3 (95%CI 1.1-1.6) per 100.000 patients among 70-74 year olds; 0.9 (95%CI 0.7-1.2) per 100,000 among 75-79 years old; 1 (95%CI 0.8-1.4) per 100,000 patients among 80-84 yearolds; 0.8 (95%CI 0.6-1.1) per 100,000 patients 85 years old and above.

Interestingly, Afro-American and Hispanic populations have a lower probability of developing testicular cancer in later years; 1 (95%CI 1.3-2.4) per 100.000 among 50-54 years old versus 5.4 (95%CI 5-5.8) per 100,000 in white for the same age group; 1 (95%CI 0.6-1.5) per 100,000 among 55-59 years old versus 3.9 (95%CI 3.6-4.3) in black and white populations respectively.

In contrast, in Caucasian patients, diagnosis of testicular cancer is recorded even in the 85 years and above group. Moreover, the long-term trends in SEER Age-Adjusted incidence rates between 1975 and 2018, showed an increasing incidence of testicular cancer between the ages of 50-64, whereas above 65 the incidence was stable over this period.

**Figure 1:**
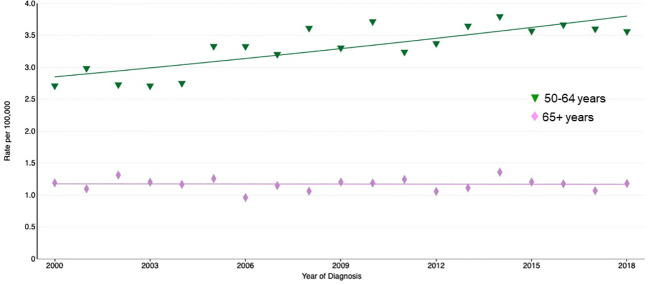
Recent trends in SEER age-adjusted incidence rates, 2000-2018.

Focusing on more recent trends in SEER Age-adjusted incidence rates (between 2014-2018), although the group above 65 years old appears stable, the incidence of testicular tumors slightly increased in 65- to 74-year-olds, but slightly decreased after 75 (**Figure 1**).

In males aged 0-49 years, only 217 individuals have to be followed throughout their lives in order to detect a testicular tumor; this number increases to 2084 in the age range 50-69 and 4652 in the range 70-84 (1), which demonstrates the rarity of this cancer in older populations.

The oldest patient in the literature was a remarkable case of pure embryonal carcinoma in an Afro-American of 96 years (8).

In a survey from forty-one US cancer registries for the years 1999-2014, 9353 seminomas, 2227 non-seminomas, 533 spermatocytic tumors, 4534 lymphomas, 288 sex cord stromal tumors were diagnosed in men aged 50 years and older (9). The majority of non-seminoma were mixed germ cell tumors (1339) followed by embryonal carcinoma (426). Regarding seminoma, 74% of the patients were aged 50-59, 18% were 60-69, 6% 70-79, and the few remaining were older than 80. For non-seminoma, 76% were aged 50-59, 17% were 60-70, 6% were 70-80 and only 1%, or less, above 80.

In a smaller review in the UK among 50 cases of GCT diagnosed at the age of 60 years and above (average age was 67), the majority was seminoma (82%) and only 2 cases had pure non-seminoma (embryonal carcinoma). Vascular invasion was seen in 18 cases (36%), while rete testis invasion in 70%. Eleven cases were T1. As far as tumor size is concerned, median was 6 cm (6).

In one of the few studies addressing this population (over 50 years of age) from the Memorial Sloan Kettering Cancer Centre (10), the percentage of non-seminoma was 34.7% and seminoma 62.7%. Altogether, out of nearly 4000 patients observed at this high-volume referring center, only 5.6% were above 50 years of age at diagnosis and primary mediastinal germ cell cancer was rare (0.3%).

In a large scale-study from the Cancer Registration Committee of the Japan (11), 11% of 1119 patients have been diagnosed with primary testicular cancer above 50 years of age, and the incidence of seminoma in elderly patients was reported as significantly higher (75%) than the younger population (61%).

## Differences in stage and outcome between young and elderly patients

Data from the population-based Cancer Registry in the Netherlands (12), indicate that patients above the age of 50 years have worse disease-specific survival. The 5-year relative survival rate is approximately 94% in men younger than 50 years versus 74% in those older than 50 years (all stages).

The same Dutch authors, using the data from the SEER Program of the National Cancer Institute in the USA, analysed 12,811 patients with testicular cancer. Nearly 93% were younger than 50 years of age (nearly 50% each with seminoma and non-seminoma). Eight percent of the study population was older than 50 years (8) and the majority of these cases was seminoma (72%).

Interestingly, patients who developed a germ-cell tumor before the age of 50 years had better 10-year relative survival than those who developed one after the age of 50 (90.8% versus 84%). The largest difference in survival was seen within the first year after diagnosis.

When stratifying by histology, the difference in relative survival remained. When stratifying by stage no difference was observed in the 10-year relative survival between younger and older patients in localized (Stage 1) seminoma (97.9% versus 98%). When analysing non-seminoma (Stage 1) cases, a significant difference was observed (95.1% versus 88.9%).

In advanced patients, the 10-year relative survival was markedly better for patients< 50 years than their older counterparts: 89.7% versus 69.6% for seminoma and 76.9% versus only 57% for non-seminoma (**Table 1**).

**Table 1:** Summarizes the main characteristics of germ cell tumors when diagnosed in “elderly” patients.

**Incidence /100.000 (according to age)**	50-54 yrs: 4.6 55-59 yrs: 3.4 60-64 yrs: 2.3 65-69 yrs: 1.5 ≥ 70 yrs: ≤ 1
**Prevalent Histology**	Non Seminoma
**10 –yrs survival Stage I**	Seminoma=98% Non Seminoma=89%
**10-yrs survival in Advanced Disease**	Seminoma=77% Non Seminoma=57%
**1^ line therapy**	BEP remains the preferred option

Again, the major difference was observed in the first year after diagnosis. Theprevious data from SEER take into consideration patients treated from 1973 until 1997, so those treated before the widespread use of cisplatin combinations may have received less intensive or less appropriate treatment (9).

When the Authors re-analysed the data taking into consideration patients > 50 years treated after 1985 (when cisplatin was universally employed), relative survival was in fact better than for those patients treated before 1985, but nevertheless the difference remained.

More recent data from England and Wales (13) confirm the poorer prognosis of patients older than 50 years of age. Even if survival rose substantially for older men during the 1990s and the decline in survival became less marked, the 5-year survival was over 90% for men diagnosed under the age of 50, but less than 70% for men aged 70-79 years (all stages included). In the Japanese study (11) despite the fact that elederly patients have been diagnosed in general in good prognosis group (84% versus 53%) cancer-specific survival was not superior compared to the one of patients < 50years.

The possible causes of reduced survival rates in patients with germ cell tumors > 50-60 years is a matter of concern.

Very few such patients have been accrued in clinical trials in the last decades also due to the rarity of such cases (9), so detailed clinical characteristics and treatment tolerability and outcome are lacking.

## Treatments and related toxicities

We have no information in the elderly population regarding retroperitoneal lymph node dissection in such patients from large mono-institutional series or reviews (14, 15). However, such procedure is recommended in a limited number of GCT patients, according to guidelines.

As far as chemotherapy is concerned, the BEP schedule has become the standard medical treatment for the last few decades (16), consisting of cisplatin, etoposide and bleomycin. All three drugs have been shown to be associated with increased toxicity in the elderly (17), and this raises the possibility that elderly patients may have received a modified, reduced dose to counter high toxicity and /or have been left untreated for fear of treatment-related toxicity (18, 19).

All these publications dealt with elderly patients in general, so leaving an open question mark regarding “relatively elderly” germ cell cancer patients, and again the evaluation of the effect of chemotherapy in older patients with testicular cancer is hampered because they are rarely included in clinical trials.

In a recent paper on good risk patients, some were above the age of 50, but the median age was 30 years, and no specific data on elderly patients were reported (20).

Virtually no data exist on patients older than 50 years receiving second or subsequent line therapy (refractory/relapsing patients).

There is some data from the European Society for Blood and Marrow Transplantation (EBMT) on 234 patients aged 50-59 years and on 26 patients aged 60-69 years who underwent high-dose chemotherapy and stem cell rescue at relapse (21).

In these patients, treatment-related mortality was dependent on the high-dose regimen rather than other characteristics, the best tolerated schedule being carboplatin and etoposide. The death rate due to treatment was below 4%.

A recent report from Indiana University on 116 relapsing germ cell tumor patients > 40 years at initiation of high-dose chemotherapy (median age 40.1 to 70.5 years) showed similar outcome and toxicity in < 40 and > 40 years, with toxic death rate around 3% (22).

The largest mono-institutional series of 50 patients older than 50 years treated with first line (mainly EP or BEP or carboplatin and etoposide in a minority of cases) chemotherapy, has been published by Memorial Sloan Kettering Cancer Centre (10) including 236 such patients (62% seminoma).

The 5-year actuarial overall survival for patients diagnosed < 50 years was 0.89 and 0.82 for those in the older age category. Thirty-eight of these patients have died, 23 of germ cell tumors, 5 of second non-germ cell cancer and 10 from non-cancer causes.

Treatment-related complications were frequent among all regimens. In 60% of patients treatment had to be discontinued or shifted to another regimen or delivered with a delay of more than seven days. Interestingly, 22 (44%) patients experienced at least one episode of febrile neutropenia, in six despite the use of primary prophylaxis with G-CSF from cycle one. The authors recommend anyway the use of granulocyte colony stimulating factors (G-CSF or pegylated G-CSF) in all patients with advanced germ cell cancer in first line (10).

Advanced age has also been identified as a predictive factor for developing febrile neutropenia in an Austrian study (23), together with poor performance status and poor-risk.

Age-related changes in organ function influence class-specific toxicity, and hematopoietic reserve is also thought to decrease with age. Moreover, it should be kept in mind that all regimens employed in treatment of germ cell tumors (BEP or EP mainly) are used in a population of patients usually younger than 40 -45 years of age. In some centres (24), the policy is to use prophylactic dose attenuation (i.e., delivering just one dose of bleomycin, or substituting carboplatin to cisplatin or reducing the doses of etoposide). At present, however, the acceptable results obtained in these patients with current treatment regimes, and the rarity of these tumors in older age cohorts, means that there is insufficient evidence to justify any dose reduction.

In a recent report by the International Germ Cell Cancer Collaborative Group (IGCCG-Update Consortium) (25) age has for the first time been included among the negative prognostic factors together with the presence of lung metastases. Each decade translates into a 25% increase in the risk of progression.

Very limited data are available in the adjuvant setting in this patient population (26) where feasibility has been reported also in patients over 70. A single course of adjuvant chemotherapy, either carboplatin for seminoma or BEP for non-seminoma, can be proposed in well informed patients, also considering the much more aggressive treatment (and maybe not feasible) that would be required in case of recurrence.

## Spermatocytic tumors (formerly spermatocytic seminoma)

This term was adopted in 2016 as a replacement of spermatocytic seminoma (3). In fact, this entity has no correlation with seminoma: no *in situ* neoplasia is apparent, as it does not originate from *in situ* germ cells, no extragonadal disease is seen, there are no cases in females, no chromosomal alterations typical of other germ cell tumors are present (e.g., isochromosome 12p), and it presents with a negative tumor marker profile (4). It originates from the germ cell lineage, has a very rare potential for metastatic disease (25), and has never been reported in combination with other germ cell types. It is rare (nearly 1% of all germ cell tumors), and is more frequently bilateral compared to seminoma. Nevertheless, it is the most frequent germ cell tumor in more advanced ages: median age is in the sixth decade (25) and it is extremely rare in young adolescents. Nearly 70% are reported on patients over 40 years of age.

While the incidence of seminoma and non seminoma decline after 50 years, the incidence of spermatocytic tumors increases steadily (9).

Vascular invasion is present only in a minority of cases (27, 28) and median maximum diameter is around 5 cm. The prognosis is rather good except for rare sarcomatous transformations. The sometime used term “anaplastic spermatocytic tumor” does not carry a worse prognosis and generally these tumors are very indolent. In the rare event of metastasis to the retroperitoneal lymph nodes they are treated with radiation therapy. Generally, orchidectomy is enough for the vast majority of these tumors which are considered as benign in the vast majority of cases.

In the event of metastatic disease and when surgery is not or no more an option, chemotherapy (BEP or BEP-like) has been used but with poor results, from the few reported cases in the literature (26).

## Discussion

Elderly patients are considered in our review those patients > 50 years of age. From the few data in the literature from different geographical areas, it is clear that the majority of such patients have a diagnosis of seminoma, generally with better stage categorization (i.e. good prognosis risk), while non-seminoma or mixed tumors are rarer in this population. The percentage of elderly patients is around 6-11% of the whole testicular germ cell incidence, with declining number of cases in subsequent decades. Seminoma has a similar prognosis that the younger counterpart in Stage I, while Stage I non-seminoma seems to have poorer survival rate. In metastatic disease the difference is even more striking with just half of non-seminoma patients alive at 10 years or more.

The reason for such a discrepancy in outcome has not fully understood and the reduced intensity chemotherapy regimens in elderly population, may have a role (11).

The International Guidelines do not address the topic of treatment of elderly germ cell tumors, but just take into consideration germ cell tumors as a whole; the recent report by the IGCCCG Updated Consortium has anyway included age as a prognostic factor itself in non-seminoma, but no specific recommendation regarding treatment have been presented (25).

The use of granulocyte colony stimulating factors appears to be mandatory in these patients as suggested by some Authors (10).

Very few data exit on adjuvant treatment in these patients (29), and the decision has to be taken according to patient choice and comorbidities, and virtually no data are available on managing residual disease.

## Conclusions

Elderly patients may represent a challenge for medical oncologists, due to the rarity of the disease in this cohort, and to the possible toxicity of the active drugs.

Nevertheless excellent results can be achieved in this population (11) when proper drugs doses and timing are maintained.

In early stages, personalized approaches such as preference for full dose single course adjuvant BEP in high-risk non-seminoma, compared to surveillance, may be preferred considering that three or four courses of BEP in refractory disease, may have an increased risk of toxicity.

We think that age per se has not to be considered as an insuperable obstacle in order to give these patients the possibility to gain the same exceptional results, obtained in younger patients with the same disease.

## Author contributions

GR, PP, SS, and FN contributed to conception and design of the study. DA collected the data. GR wrote the first draft of the manuscript. SS, FN, PP, OC, AT, and LJRN wrote sections of the manuscript. All authors contributed to manuscript revision, read, and approved the submitted version

## Conflict of interest

The authors declare that the research was conducted in the absence of any commercial or financial relationships that could be construed as a potential conflict of interest.

## Publisher’s note

All claims expressed in this article are solely those of the authors and do not necessarily represent those of their affiliated organizations, or those of the publisher, the editors and the reviewers. Any product that may be evaluated in this article, or claim that may be made by its manufacturer, is not guaranteed or endorsed by the publisher.
